# Current Gaps in Point‐of‐Care Ultrasound Supervision in Generalist Practice in Japan: A Nationwide Survey

**DOI:** 10.1002/jgf2.70180

**Published:** 2026-09-18

**Authors:** Toru Yamada, Takuma Kimura, Kyoko Shigetomi, Takahiro Shinohara, Shuji Ouchi, Suguru Mabuchi, Hiroyuki Ichige, Toru Kameda, Shumpei Yoshino, Yuka Kitano, Keita Endo, Kazuya Okada, Hiroshi Kamijo, Hiromizu Takahashi, Tomoko Kusama, Takeshi Ishida, Masayoshi Hashimoto

**Affiliations:** ^1^ Department of General Medicine, Graduate School of Medical and Dental Sciences Institute of Science Tokyo Tokyo Japan; ^2^ Department of R&D Innovation for Home Care Medicine, Graduate School of Medical and Dental Sciences Institute of Science Tokyo Tokyo Japan; ^3^ Department of Cardiovascular Surgery Juntendo University Hospital Tokyo Japan; ^4^ Department of Community Medicine (Ibaraki), Graduate School of Medical and Dental Sciences Institute of Science Tokyo Tokyo Japan; ^5^ Department of Ultrasound Medicine Saiseikai Utsunomiya Hospital Tochigi Japan; ^6^ Department of Intensive Care Medicine Aso Iizuka Hospital Iizuka Fukuoka Japan; ^7^ Emergency and Critical Care Medicine, School of Medicine St. Marianna University Kawasaki Kanagawa Japan; ^8^ Department of Nephrology, Diabetes and Endocrinology Tokyo Bay Urayasu Ichikawa Medical Center Chiba Japan; ^9^ Department of Critical Care Medicine Tokyo Metropolitan Bokutoh Hospital Tokyo Japan; ^10^ Department of Emergency and Critical Care Medicine Shinshu University Hospital Nagano Japan; ^11^ Department of General Medicine Juntendo University Urayasu Hospital Chiba Japan; ^12^ Oita University of Nursing and Health Sciences Oita Japan

**Keywords:** clinical supervision, generalist practice, nurse, nurse practitioner, online supervision, point‐of‐care ultrasound

## Abstract

**Background:**

Point‐of‐care ultrasound (POCUS) is increasingly used in generalist practice by physicians, nurse practitioners, and nurses. However, the current availability of clinical supervision and preferred supervision models for POCUS remains unclear.

**Methods:**

We conducted an anonymous web‐based survey among members of five academic societies related to generalist practice in Japan between April 2024 and March 2025. The target respondents were physicians, nurse practitioners, and nurses. The survey assessed POCUS use in daily clinical practice, current supervision status, preferred supervision models, and previous training experience.

**Results:**

A total of 1056 valid responses were analyzed, including 835 physicians and 221 nurses or nurse practitioners. Overall, 735 respondents (69.6%) reported performing some form of POCUS in daily clinical practice. The most common current supervision status was having no available supervisor inside or outside the facility (36.9%), more common among physicians than among nurses and nurse practitioners (42.2% vs. 17.2%, *p* < 0.001). Real‐time online supervision was reported as the current supervision status by 2.1% of respondents, whereas 56.3% selected it as a preferred model and 79.5% selected continuous in‐person supervision; the two items used different response formats (single choice vs. multiple response) and are not directly comparable. Fewer than 40% of respondents had received previous POCUS training, although 77.8% expressed future training needs.

**Conclusions:**

POCUS is widely used in generalist practice in Japan, but clinical supervision remains insufficient. Reported current supervision differed from preferred supervision models, suggesting that real‐time online supervision may be a feasible strategy to support safe and sustainable POCUS implementation.

**Abbreviations**
CCM: critical care medicine
EM: emergency medicine
FOCUS: focused cardiac ultrasound
IM: internal medicine
JAHCM: Japanese Association for Home Care Medicine
JPCA: Japan Primary Care Association
JSHGM: Japanese Society of Hospital General Medicine
JSIM: Japanese Society of Internal Medicine
JSNP: Japan Society of Nurse Practitioners
POCUS: point‐of‐care ultrasound

## 1 Background

In recent years, point‐of‐care ultrasound (POCUS) has increasingly been recognized as a tool that contributes to improved diagnostic accuracy and procedural safety. Its use has expanded in emergency medicine, primary care, general medicine, and home medical care settings [1, 2, 3, 4]. POCUS supports clinical decision‐making in situations with limited time and resources, and its appropriate use has the potential to improve patient outcomes. These characteristics are particularly relevant to generalist practice, where clinicians manage a broad range of clinical problems across outpatient, inpatient, and home care settings. With the increasing availability of portable ultrasound devices, POCUS is now used by a variety of healthcare professionals beyond traditional specialty boundaries. The utility of task shifting POCUS‐related roles to non‐physician providers, such as nurse practitioners and nurses, is also becoming increasingly evident [5, 6, 7, 8]. In low‐ and middle‐income countries, where specialists and diagnostic equipment may be limited, combining task shifting with POCUS may help address gaps in diagnostic capacity [9]. As POCUS use expands across settings and professions, systems to ensure safe implementation and quality assurance have become increasingly important.

Appropriate use of POCUS requires structured training and clinical supervision. Despite growing demand, major educational challenges remain, including limited training opportunities, insufficient numbers of qualified instructors, and the absence of standardized curricula [10, 11, 12, 13, 14]. The shortage of qualified instructors is a major issue for both physicians and nurses or nurse practitioners [10]. In Japan, some academic societies have begun offering POCUS training and certification for physicians, but unified national qualification requirements and standardized training systems have not yet been established [15]. POCUS has also not been incorporated into formal educational curricula as a certification requirement for nurses and nurse practitioners, and the current status of their training and supervision remains unclear. Globally, POCUS training programs and educational objectives for nurses and nurse practitioners are still developing [6, 16]. Although training is an essential first step, supervision is also needed to support image acquisition, image interpretation, and integration of ultrasound findings into real‐time clinical decision‐making. This is especially important in generalist settings, where access to ultrasound specialists or experienced POCUS instructors may be limited.

Our previous nationwide survey described POCUS utilization, barriers, and facilitators among physicians, nurse practitioners, and nurses in Japan [10]. However, the availability of clinical supervision, current use of remote or online supervision, and preferences for different supervision models were not the focus of that study. Therefore, this study aimed to examine the current status of POCUS supervision and preferred supervision models among physicians, nurse practitioners, and nurses in Japan, with particular attention to the gap between current supervision systems and preferred models of real‐time in‐person or online supervision.

## 2 Methods

### 2.1 Participants and Setting

An anonymous web‐based survey was conducted among members of five major academic societies related to generalist practice in Japan: the Japan Primary Care Association (JPCA), the Japanese Society of Hospital General Medicine (JSHGM), the Board of Certified Committee of the Japanese Society of Internal Medicine (JSIM), the Japanese Association for Home Care Medicine (JAHCM), and the Japan Society of Nurse Practitioners (JSNP), which is the primary academic society for nurse practitioners in Japan. Data were analyzed from surveys conducted between April and June 2024 among members of JPCA, JSHGM, JAHCM, and JSNP [10], and from a similar survey conducted between January and March 2025 among members of the Board of Certified Committee of JSIM. The April–June 2024 dataset was previously used to report POCUS utilization, barriers, and facilitators [10]. In contrast, the present study focuses on clinical supervision for POCUS, including supervisor availability, current use of remote or online supervision, and preferred supervision models, and additionally includes physician respondents from JSIM.

JPCA, JSHGM, and JAHCM are primarily physician‐oriented societies, although their membership also includes other healthcare professionals such as nurses. JSHGM is the largest academic society in Japan for hospitalists, and JPCA is the largest primary care society in Japan, including both family physicians and hospitalists. The Board of Certified Committee of JSIM consists of physicians who hold the Fellow of the Japanese Society of Internal Medicine designation within the largest internal medicine society in Japan. JAHCM was included because of the relevance of POCUS in home medical care. Together, these societies were considered appropriate for investigating the POCUS supervision environment in generalist practice, including outpatient, inpatient, emergency, and home care settings.

The survey was distributed through the mailing lists of the participating societies. As of April 2024, the numbers of registrants were 7257 for JPCA (including 6265 physicians), 2280 for JSHGM (including 11 nurses), 3463 for JAHCM (including 3340 physicians), and 736 for JSNP. The mailing list of the Board of Certified Committee of JSIM included approximately 5000 registrants, as reported by the society secretariat. The simple total of these mailing lists was approximately 18,700 registrants.

### 2.2 Survey Questionnaire

The questionnaire was developed by the co‐authors affiliated with the Department of General Medicine, Institute of Science Tokyo. Because no validated instrument was available for this purpose, the items were not derived from a formal literature review but were drafted on the basis of the responses to post‐course questionnaires from a nationwide POCUS training course previously organized by the authors [15], which reflected the practical concerns raised by participating clinicians regarding POCUS use, training, and supervision. The draft was reviewed and revised through iterative discussion among the authors until consensus was reached, and was then pilot tested with five physicians. No items were reported as unclear or ambiguous, and the mean time to completion was 7 min. The same questionnaire was used in the survey previously reported [10].

The survey was conducted anonymously using a web‐based platform. The target respondents were physicians, nurse practitioners, and nurses who were members of any of the five target academic societies. Responses were excluded if participants did not consent to the study, belonged to professions outside the target groups, or did not complete the survey. Because respondents may have belonged to multiple societies and the extent of membership overlap was unknown, only one response per participant was included, and duplicate responses were excluded.

**FIGURE 1:**
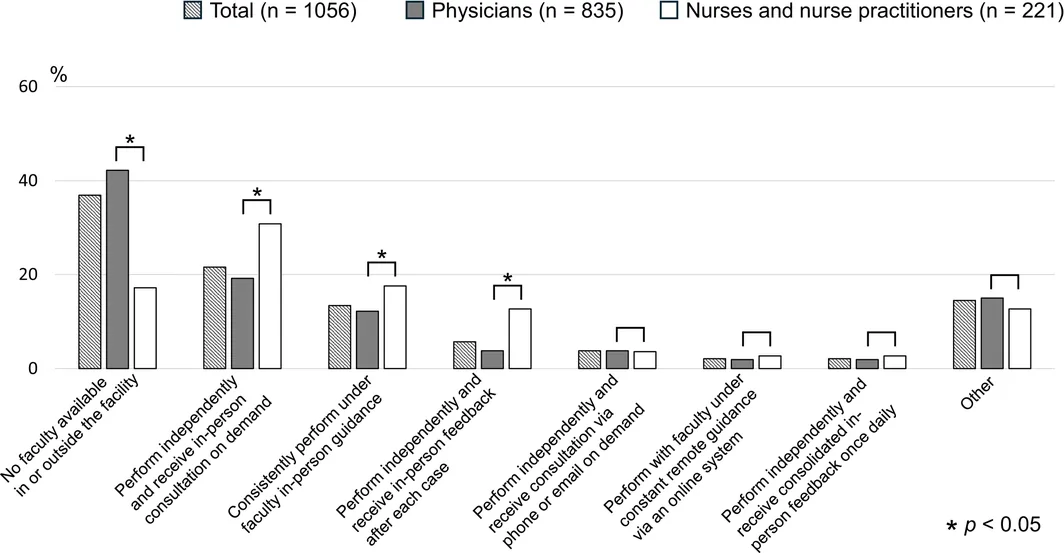
Comparison of current POCUS supervision status between physicians and nurses/nurse practitioners. The response “A faculty available but supervision not provided” was combined with “Other.”

The questionnaire included items on respondents' institutional affiliation, clinical specialty, practice setting, years of POCUS experience, frequency of use, perceived barriers and facilitators, current supervision environment, and preferred supervisory methods. In the present study, we focused on items related to the current status of clinical supervision for POCUS and preferred supervision models. Training experience and future training needs were analyzed as contextual variables to help interpret the supervision environment. The survey items and their response formats are shown in Table 1. The item on the current status of POCUS supervision (item 34) was a single‐choice question with nine response options, comprising six supervision models, “No faculty available in or outside the facility,” “A faculty available but supervision not provided,” and “Other.” The item on the preferred status of POCUS supervision (item 35) was a multiple‐response question comprising the same six supervision models, in which respondents could select all applicable options. In Figure 1 and File S3, the response “A faculty available but supervision not provided” was combined with “Other” for presentation.

**TABLE 1:** Survey items and response formats.

No.	Survey item	Response format
Background		
1	Are you a physician, nurse, or nurse practitioner?	Single choice
2	Is this the first time you have answered this survey?	Single choice (yes/no)
3	What is your age?	Numerical entry
4	What is your gender?	Single choice
5	What is your occupation?	Single choice
6	What year did you graduate from medical school or nursing school?	Numerical entry
7	What is the main type of facility where you work?	Single choice
8	What is the main department you are affiliated with at your workplace?	Single choice
9	Do you regularly provide medical care or nursing care to inpatients in general wards?	Single choice (yes/no)
10	Do you regularly provide medical care or nursing care to inpatients in intensive care settings?	Single choice (yes/no)
11	Do you regularly provide medical care or nursing care to outpatients?	Single choice (yes/no)
12	Do you regularly provide home care or nursing?	Single choice (yes/no)
POCUS use in daily clinical practice		
13–19	Do you perform any kind of POCUS/FOCUS/lung/vascular/abdominal/musculoskeletal/procedural ultrasound in your daily clinical practice? (asked separately for each modality)	Single choice (yes/no) for each item
POCUS training experience		
20–26	Have you ever received any form of POCUS/FOCUS/lung/vascular/abdominal/musculoskeletal/procedural ultrasound training in the past? (asked separately for each modality)	Single choice (yes/no) for each item
Future needs for POCUS training		
27–33	Would you like to receive any POCUS/FOCUS/lung/vascular/abdominal/musculoskeletal/procedural ultrasound training in the future? (asked separately for each modality)	Single choice (yes/no) for each item
Current and preferred status of POCUS supervision		
34	What is the current status of POCUS supervision in your routine clinical practice?	Single choice (nine options; see footnote)
35	What is your preferred status of POCUS supervision in your clinical practice? (the six supervision models included in item 34)	Multiple choice (six options; see footnote)

*Note:* Response options for item 34: (1) Consistently perform under faculty in‐person guidance; (2) Perform independently and receive in‐person feedback after each case; (3) Perform independently and receive consolidated in‐person feedback once daily; (4) Perform independently and receive in‐person consultation on demand; (5) Perform with faculty under constant remote guidance via an online system; (6) Perform independently and receive consultation via phone or email on demand; (7) No faculty available in or outside the facility; (8) A faculty available but supervision not provided; (9) Other. Item 35 comprised options (1)–(6), and respondents could select all applicable options.

Abbreviations: FOCUS, focused cardiac ultrasound; POCUS, point‐of‐care ultrasound.

### 2.3 Outcomes

**FIGURE 2:**
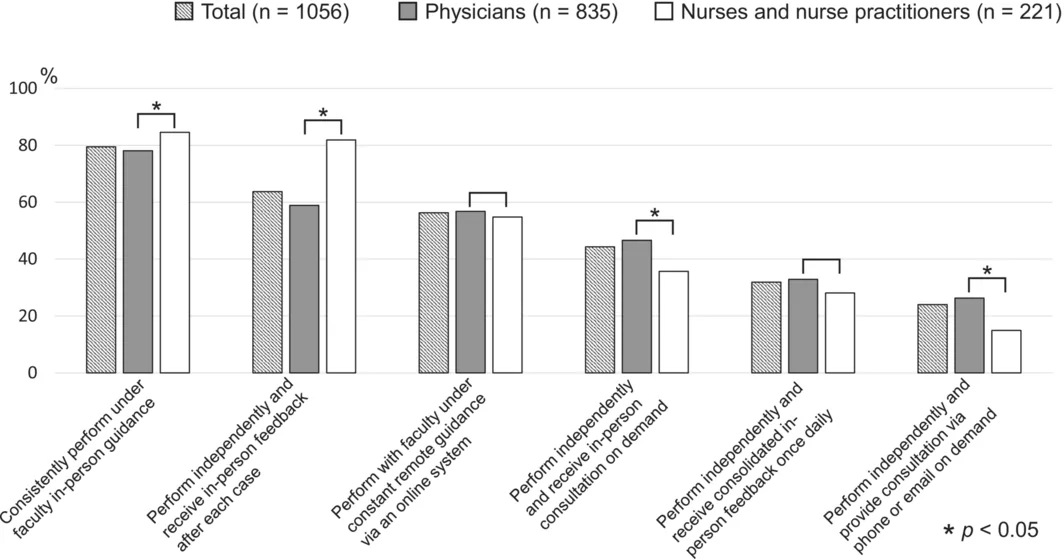
Comparison of preferred POCUS supervision models between physicians and nurses/nurse practitioners.

The primary outcomes were the current status of POCUS supervision and preferred supervision models. Current supervision status included the availability of supervisors inside or outside the respondent's institution, in‐person supervision, on‐demand consultation, remote consultation by telephone or email, and real‐time online supervision. Preferred supervision models comprised six options: continuous in‐person supervision, case‐by‐case in‐person feedback, once‐daily consolidated in‐person feedback, on‐demand in‐person consultation, remote consultation by telephone or email, and real‐time online supervision (Figure 2). Secondary outcomes included previous POCUS training experience and future needs for POCUS training.

### 2.4 Statistical Analysis

Comparisons of participant characteristics, training experience, future needs for POCUS training, current status and preferred method of POCUS supervision were conducted using the Wilcoxon rank‐sum test for continuous variables and the chi‐square test for categorical variables. Because the item on preferred supervision allowed multiple responses from the same respondent, the proportions of the different preferred supervision models were not compared statistically with one another, and differences between models are described descriptively. For the same reason, the proportions of current and preferred supervision, which were obtained using different response formats, were not compared statistically. Statistical significance was defined as a *p* value of < 0.05. All data were analyzed using STATA software version 17.0 (StataCorp LLC, College Station, TX, USA).

The question regarding the current status of POCUS supervision was presented to all respondents, including those who did not perform POCUS in daily clinical practice, in order to capture the availability of supervisors within and outside the facility regardless of current POCUS use. As a subgroup analysis, the current status of POCUS supervision was additionally analyzed among respondents who reported performing some form of POCUS in daily clinical practice (*n* = 735). In this subgroup analysis, comparisons between physicians and nurses or nurse practitioners for each supervision category were performed using Fisher's exact test.

## 3 Results

### 3.1 Participants

A total of 1093 responses were collected, of which 37 were excluded because of duplicate entries, ineligible professions, incomplete responses, or lack of consent to participate. This left 1056 responses for inclusion in the analysis, including 835 physicians and 221 nurses or nurse practitioners. The average time since graduation was 24.2 years (SD 11.4) for physicians and 21.2 years (SD 9.1) for nurses and nurse practitioners, indicating that respondents in both groups had relatively extensive clinical experience. Approximately 41% of physicians were affiliated with community hospitals and 45% with clinics, whereas 56% of nurses and nurse practitioners worked in community hospitals and 25% in clinics. Physicians were most commonly specialists in family medicine (41%), followed by hospital medicine (32%), whereas nurses and nurse practitioners were most commonly affiliated with family medicine (23%), followed by emergency medicine/critical care and surgical specialties, each at 20%.

Overall, 735 respondents (69.6%) reported performing some form of POCUS in daily clinical practice. Physicians had higher usage rates for vascular, abdominal, and musculoskeletal ultrasound (all *p* < 0.05), whereas no significant differences were observed between groups for focused cardiac ultrasound (FOCUS), lung ultrasound, or procedural ultrasound (Table 2).

**TABLE 2:** Participant characteristics.

	Total *n* = 1056	Physicians *n* = 835	Nurses and NPs *n* = 221	*p*
Age (years), mean (SD)	48.7 (10.7)	49.7 (11.0)	44.7 (8.3)	< 0.001
Male, *n* (%)	728 (68.9)	655 (78.4)	73 (33.0)	< 0.001
Years since graduation, mean (SD)	23.6 (11.0)	24.2 (11.4)	21.2 (9.1)	< 0.001
Institution, *n* (%)				
Clinic/home care station	429 (40.6)	374 (44.8)	55 (24.9)	< 0.001
Community hospital	468 (44.3)	345 (41.3)	123 (55.7)	< 0.001
University hospital	147 (13.9)	110 (13.2)	37 (16.7)	0.173
Other	12 (1.1)	6 (0.7)	6 (2.7)	
Department, *n* (%)				
Hospital medicine	303 (28.7)	268 (32.1)	35 (15.8)	< 0.001
Family medicine	395 (37.4)	344 (41.2)	51 (23.1)	< 0.001
IM subspecialties	176 (17.7)	145 (17.4)	31 (14.0)	0.236
EM, CCM	64 (6.1)	20 (2.4)	44 (19.9)	< 0.001
Surgical specialties	86 (8.1)	42 (5.0)	44 (19.9)	< 0.001
Others	32 (3.0)	16 (1.9)	16 (7.2)	
Clinical field, *n* (%)*				
Inpatient care	503 (47.6)	375 (44.9)	128 (57.9)	0.001
Critical care	218 (20.6)	136 (16.3)	82 (37.1)	< 0.001
Outpatient care	868 (82.2)	757 (90.7)	111 (50.2)	< 0.001
Home visit care	565 (53.5)	491 (58.8)	74 (33.5)	< 0.001
POCUS use in daily practice, *n* (%)				
Any form of POCUS	735 (69.6)	595 (71.3)	140 (63.4)	0.023
FOCUS	530 (50.2)	429 (51.4)	101 (45.7)	0.133
Lung	366 (34.7)	290 (34.7)	76 (34.4)	0.924
Vascular	434 (41.2)	361 (43.2)	73 (33.0)	0.006
Abdomen	679 (64.3)	564 (67.5)	115 (52.0)	< 0.001
Musculoskeletal	267 (25.3)	235 (28.1)	32 (14.5)	< 0.001
Procedure	491 (46.5)	379 (45.4)	112 (50.7)	0.161

Abbreviations: CCM, critical care medicine; department, primary department of physicians, nurses, and nurse practitioners; EM, emergency medicine; FOCUS, focused cardiac ultrasound; IM, internal medicine; NP, nurse practitioner.

* Multiple answer selections permitted.

### 3.2 Current Status of POCUS Supervision

The most commonly reported supervision status when performing POCUS in daily practice was “No faculty available in or outside the facility,” accounting for 36.9% overall. This status was significantly more common among physicians than among nurses and nurse practitioners (42.2% vs. 17.2%, *p* < 0.001). Among nurses and nurse practitioners, the most common response was “Perform independently and receive in‐person consultation on demand” (30.8%).

In‐person supervision was more commonly available to nurses and nurse practitioners than to physicians, both for continuous in‐person guidance (17.6% vs. 12.2%, *p* = 0.035) and on‐demand in‐person consultation (30.8% vs. 19.2%, *p* < 0.001). In contrast, the current use of remote supervision was limited. Only 3.8% of respondents reported having remote consultation available by telephone or email, and only 2.1% reported receiving real‐time online supervision, with no significant differences between physicians and nurses/nurse practitioners (*p* = 0.883 and *p* = 0.460, respectively) (Figure 1). In a subgroup analysis restricted to the 735 respondents who reported performing POCUS in daily clinical practice, the most common supervision status remained “No faculty available in or outside the facility” (30.9%), which was again significantly more common among physicians than among nurses and nurse practitioners (36.1% vs. 8.6%, *p* < 0.001). The current use of real‐time online supervision remained limited to 1.2% of respondents. These findings were consistent with those of the overall analysis (File S3).

### 3.3 Preferred Supervision Models

The most preferred supervision model was continuous in‐person supervision, selected by 79.5% of respondents. This preference was significantly higher among nurses and nurse practitioners than among physicians (84.6% vs. 78.1%, *p* = 0.033). The second most preferred model was independent performance followed by case‐by‐case in‐person feedback, which was also more frequently selected by nurses and nurse practitioners than by physicians (81.9% vs. 58.9%, *p* < 0.001).

Real‐time online supervision was the third most preferred model overall, selected by 56.3% of respondents. This proportion was higher than that for on‐demand in‐person consultation after independent performance, which was selected by 44.3% of respondents. Preferences for real‐time online supervision were similar between physicians and nurses/nurse practitioners (56.8% vs. 54.8%, *p* = 0.597) (Figure 2).

### 3.4 Current and Preferred Supervision Environments

Because current supervision status was assessed with a single‐choice item and preferred supervision models with a multiple‐response item, the proportions obtained from the two items are not directly comparable and are therefore described descriptively. Real‐time online supervision was reported as the current supervision status by 2.1% of respondents, whereas 56.3% selected it as a preferred model. Similarly, continuous in‐person supervision was reported as the current supervision status by 13.4% of respondents and was selected as a preferred model by 79.5%. These findings suggest that respondents preferred more structured and real‐time supervision than they reported receiving in daily clinical practice.

### 3.5 POCUS Training Experience and Future Training Needs

As contextual information, 37.7% of respondents had received some form of POCUS training in the past. This proportion was significantly higher among nurses and nurse practitioners than among physicians (52.5% vs. 33.8%, *p* < 0.001) (File S1). Overall, 77.8% of respondents expressed a desire to receive POCUS training in the future, with a significantly higher proportion among nurses and nurse practitioners than among physicians (89.1% vs. 74.9%, *p* < 0.001) (File S2).

## 4 Discussion

In this nationwide survey of physicians, nurse practitioners, and nurses who were members of five generalist‐related academic societies in Japan, approximately 70% of respondents reported performing some form of POCUS in daily clinical practice. However, the supervision environment appeared insufficient. The most common supervision status was the absence of a supervisor both inside and outside the facility, particularly among physicians. Although continuous in‐person supervision was the most preferred model, more than half of respondents also preferred continuous online supervision, which was more frequently selected than on‐demand in‐person supervision. In contrast, real‐time online supervision was rarely reported as the current supervision status. Although the two items used different response formats and their proportions are not directly comparable, these findings suggest a discrepancy between the current status of POCUS supervision and the preferred supervisory environment.

### 4.1 Current Status of POCUS Supervision

In daily clinical practice, the most commonly reported supervision status was the absence of a supervisor both inside and outside the facility. This was reported by 36.9% of respondents overall and was more common among physicians than among nurses and nurse practitioners. In Japan, physicians are permitted to perform ultrasound examinations without restrictions on the target organ, regardless of whether they hold board certification in a relevant specialty or have formal ultrasound training. Although there are nearly 350,000 physicians in Japan [17], fewer than 3000 are board‐certified ultrasound specialists [18]. It is therefore likely that many physicians performing POCUS do so without certification as ultrasound specialists or regular access to expert supervision.

The proportion of respondents without available supervisors was lower among nurses and nurse practitioners than among physicians. This may be partly because nurses and nurse practitioners in Japan are permitted to perform ultrasound examinations under physician supervision. However, there are no specific requirements regarding their POCUS training, the types of ultrasound examinations they may perform, or the qualifications of the supervising physician. Therefore, even when a supervising physician is formally present, that physician may not necessarily be able to provide POCUS‐specific instruction. In our survey, 17.2% of nurses and nurse practitioners reported no available supervisor either inside or outside their facility, suggesting an “effective absence of supervision” for POCUS.

POCUS is a simplified form of ultrasound primarily intended to support bedside screening and clinical decision‐making. This makes it suitable for use by a wide range of healthcare professionals. In medically underserved areas, such as remote or island regions where physicians and diagnostic infrastructure are limited, the use of POCUS by non‐physician healthcare professionals may strengthen local diagnostic capacity [9]. However, broader use of POCUS must be accompanied by systems that maintain examination quality. Inadequate skills may lead to misinterpretation or inappropriate clinical decisions, making training, supervision, and competency standards essential for safe implementation.

### 4.2 In‐Person and Online Supervision

Continuous in‐person supervision was the most preferred supervision model. This is understandable because direct supervision allows immediate feedback on probe handling, image acquisition, image interpretation, and clinical integration. However, the availability of such supervision was limited in daily practice. Notably, continuous online supervision was selected as a preferred model by 56.3% of respondents, whereas only 2.1% reported real‐time online supervision as their current supervision status. Although these proportions were obtained using different response formats and cannot be compared directly, this contrast suggests that many respondents recognized the value of real‐time supervisory support but lacked access to a feasible supervision system.

The preference for real‐time online supervision may reflect the clinical nature of POCUS. POCUS is a diagnostic approach that requires real‐time interpretation in the clinical context [1]. Unlike comprehensive ultrasonography, which is typically performed as a scheduled examination, POCUS is often used in dynamic bedside situations where clinical conditions may change rapidly. On‐demand in‐person consultation may be useful in some situations, but it may not provide timely support when immediate decision‐making is required. Online supervision could allow experienced instructors outside the facility to support image interpretation and clinical decision‐making at the point of care.

Efforts to incorporate online education into POCUS training have increasingly been proposed, particularly since the COVID‐19 pandemic [19]. These initiatives have been shown to reduce preparation time for educators and have received favorable evaluations in terms of image acquisition skills, image interpretation skills, and learner satisfaction. A randomized controlled trial comparing online education with hands‐on in‐person training for ocular POCUS found that the online approach was not inferior [20]. However, reviews of online POCUS education suggest that although it can improve knowledge and image interpretation, evidence for improving image acquisition skills remains inconsistent [21]. These findings suggest that online supervision may be most effective when combined with initial hands‐on training and used to support ongoing image interpretation, confidence building, and clinical decision‐making rather than replacing in‐person training entirely.

### 4.3 POCUS Training Experience and Future Needs

Although the present study focused primarily on supervision, training experience provides important context for interpreting the supervision gap. Fewer than 40% of respondents had received formal POCUS training, despite nearly 70% reporting POCUS use in daily practice. This mismatch suggests that many clinicians may be performing POCUS without sufficient structured educational preparation. Limited access to ultrasound equipment and lack of training opportunities have been identified as major barriers to POCUS implementation [10, 11, 12, 13, 14], and our findings are consistent with these reports.

Nurses and nurse practitioners were more likely than physicians to have received POCUS training, but they also expressed greater future training needs. Multiple studies have reported the utility of POCUS performed by nurse practitioners and nurses [5, 6, 7, 8], but the development of training programs and educational objectives for these professionals remains at an early stage [6, 16]. In Japan, POCUS has not yet been incorporated into formal curriculum requirements for nurse practitioners or nurses. The lack of training opportunities has been reported as a significant barrier to POCUS implementation for these professionals [10]. Our findings suggest that future educational systems should not only expand training opportunities but also incorporate supervision mechanisms that support safe clinical use after training.

### 4.4 Implications for Generalist Practice

These findings have important implications for generalist practice. POCUS is increasingly used in outpatient, inpatient, emergency, and home care settings, but access to ultrasound specialists or experienced POCUS instructors is often limited. Simply increasing the number of short training courses may not be sufficient to ensure safe implementation. A more sustainable model may require a combination of initial hands‐on training, competency‐based assessment, image review, case‐based feedback, and access to real‐time supervision when needed. Online supervision may be a practical strategy for connecting generalist clinicians with experienced instructors across institutional and geographic boundaries. As POCUS use expands beyond physicians to include nurses and nurse practitioners, such systems may also support interprofessional implementation while maintaining quality and safety.

## 5 Limitations

This study had several limitations. First, the survey was distributed through the mailing lists of five academic societies, with a simple total of approximately 18,700 registrants, and the 1056 analyzed responses corresponded to approximately 5.6% of this figure. However, because an unknown proportion of individuals were registered on more than one mailing list, this value does not represent an accurate response rate. The response rate was presumed to be low, and the findings may not reflect the overall status of POCUS among members of these societies. Second, participation was voluntary, and respondents may have had a relatively high interest in POCUS, which may limit generalizability. In particular, the higher training rate among nurses and nurse practitioners than among physicians may reflect response bias toward individuals who were already interested in or had received training in POCUS. Third, the survey relied on self‐reported data, and we could not objectively verify participants' POCUS skills, the quality of training received, or actual supervision practices. Fourth, although this study focused on supervision, it did not evaluate whether different supervision models were associated with improved diagnostic accuracy, procedural safety, or patient outcomes. Future studies should examine the effectiveness of in‐person and online supervision models in real clinical settings. Fifth, current supervision status was assessed with a single‐choice item, whereas preferred supervision models were assessed with a multiple‐response item. Respondents who had access to more than one form of supervision could select only one option for the current status, and the absolute proportions of current and preferred supervision are therefore not directly comparable; the differences described in this study should be interpreted descriptively.

## 6 Conclusion

This survey showed that many physicians, nurse practitioners, and nurses in generalist‐related settings in Japan use POCUS in daily clinical practice, but clinical supervision remains insufficient. The low proportion of respondents who reported real‐time online supervision as their current supervision status, together with the high proportion who selected it as a preferred model, suggests that online supervision may be a feasible strategy to support safe and sustainable POCUS implementation, particularly in settings with limited access to on‐site instructors.

## Author Contributions

**Toru Yamada:** conceptualization, methodology, investigation, formal analysis, supervision, writing – original draft, writing – review and editing, project administration, resources, data curation, visualization, validation. **Takuma Kimura:** writing – review and editing, validation, conceptualization, supervision, resources, investigation. **Kyoko Shigetomi:** conceptualization, methodology, writing – review and editing, resources, investigation. **Takahiro Shinohara:** methodology, conceptualization, investigation, resources, writing – review and editing. **Shuji Ouchi:** conceptualization, methodology, investigation, resources, writing – review and editing. **Suguru Mabuchi:** conceptualization, methodology, investigation, resources, writing – review and editing. **Hiroyuki Ichige:** conceptualization, methodology, investigation, resources, writing – review and editing. **Toru Kameda:** conceptualization, methodology, investigation, resources, writing – review and editing. **Shumpei Yoshino:** conceptualization, methodology, investigation, writing – review and editing, resources. **Yuka Kitano:** conceptualization, methodology, investigation, resources, writing – review and editing. **Keita Endo:** conceptualization, methodology, investigation, writing – review and editing, resources. **Kazuya Okada:** conceptualization, methodology, investigation, resources, writing – review and editing. **Hiroshi Kamijo:** conceptualization, methodology, investigation, writing – review and editing, resources. **Hiromizu Takahashi:** conceptualization, methodology, investigation, writing – review and editing, resources. **Tomoko Kusama:** conceptualization, methodology, investigation, writing – review and editing, project administration, resources. **Takeshi Ishida:** conceptualization, methodology, writing – review and editing, project administration, resources, supervision. **Masayoshi Hashimoto:** conceptualization, methodology, supervision, writing – review and editing, project administration, resources.

## Funding

The authors have nothing to report.

## Ethics Statement

This study adhered to the guidelines of the 2013 Declaration of Helsinki and was approved by the institutional review board of the authors' institution (Approval Number: C2023‐057). All participants received a written explanation on the web and provided their consent.

## Conflicts of Interest

T. Kimura has a competing interest related to C.U.C. Inc., Japan. All other authors declare no conflicts of interest.

## Supporting information


**File S1:** Comparison of previous POCUS training experience between physicians and nurses and nurse practitioners.

**File S2:** Comparison of future needs for POCUS training between physicians and nurses and nurse practitioners.

**File S3:** Current status of POCUS supervision among respondents who reported performing POCUS in daily clinical practice (*n* = 735).

## Data Availability

The data that support the findings of this study are available from the corresponding author upon reasonable request.
