# Supplementary material for: Current Gaps in Point‐of‐Care Ultrasound Supervision in Generalist Practice in Japan: A Nationwide Survey
Source: J Gen Fam Med. 2026 Sep 18;27(5):e70180. doi: 10.1002/jgf2.70180 (PMC13589104; doi:10.1002/jgf2.70180)
Supplement: Supplementary file 1 [file JGF2-27-e70180-s002.docx]

**Supplementary file 3**

**Current status of POCUS supervision among respondents who reported performing POCUS in daily clinical practice (n = 735)**

| **Supervision status** | **Total (n = 735)** | **Physicians (n = 595)** | **Nurses and nurse practitioners (n = 140)** | **p value** |
| --- | --- | --- | --- | --- |
| No faculty available in or outside the facility | 227 (30.9) | 215 (36.1) | 12 (8.6) | <0.001 |
| Perform independently and receive in-person consultation on demand | 191 (26.0) | 132 (22.2) | 59 (42.1) | <0.001 |
| Consistently perform under faculty in-person guidance | 106 (14.4) | 84 (14.1) | 22 (15.7) | 0.596 |
| Perform independently and receive in-person feedback after each case | 53 (7.2) | 31 (5.2) | 22 (15.7) | <0.001 |
| Perform independently and receive consultation via phone or email on demand | 29 (3.9) | 23 (3.9) | 6 (4.3) | 0.810 |
| Perform with faculty under constant remote guidance via an online system | 9 (1.2) | 8 (1.3) | 1 (0.7) | 1.000 |
| Perform independently and receive consolidated in-person feedback once daily | 18 (2.4) | 12 (2.0) | 6 (4.3) | 0.129 |
| Other | 102 (13.9) | 90 (15.1) | 12 (8.6) | — |

Values are presented as n (%).

p values were calculated using Fisher's exact test comparing physicians with nurses and nurse practitioners for each supervision category. No test was performed for the “Other” category.

Categories and their order correspond to those shown in Figure 1. POCUS, point-of-care ultrasound.
